# Bioluminescence imaging of mouse monocyte chemoattractant protein-1 expression in inflammatory processes: Bioluminescence imaging of mouse MCP-1 expression

**DOI:** 10.3724/abbs.2022143

**Published:** 2022-10-13

**Authors:** Fangyang Shao, Lei Ci, Jiahao Shi, Fei Fang, Bowen Yan, Xijun Liu, Xiangyu Yao, Mengjie Zhang, Hua Yang, Zhugang Wang, Jian Fei

**Affiliations:** 1 School of Life Sciences and Technology Tongji University Shanghai 200092 China; 2 Institute of Biophysics Chinese Academy of Sciences Beijing 100101 China; 3 College of Life Sciences University of Chinese Academy of Sciences Beijing 100049 China; 4 Shanghai Engineering Research Center for Model Organisms SMOC Shanghai 201203 China

**Keywords:** bioluminescence imaging, luciferase, MCP-1, mouse, promoter

## Abstract

Monocyte chemoattractant protein-1 (MCP-1) plays a crucial role in various inflammatory diseases. To reveal the impact of MCP-1 during diseases and to develop anti-inflammatory agents, we establish a transgenic mouse line. The firefly luciferase gene is incorporated into the mouse genome and driven by the endogenous *MCP-1* promoter. A bioluminescence photographing system is applied to monitor luciferase levels in live mice during inflammation, including lipopolysaccharide-induced sepsis, concanavalin A-induced T cell-dependent liver injury, CCl _4_-induced acute hepatitis, and liver fibrosis. The results demonstrate that the luciferase signal induced in inflammatory processes is correlated with endogenous MCP-1 expression in mice. Furthermore, the expressions of *MCP-1* and the luciferase gene are dramatically inhibited by administration of the anti-inflammatory drug dexamethasone in a septicemia model. Our results suggest that the transgenic MCP-1-Luc mouse is a useful model to study MCP-1 expression in inflammation and disease and to evaluate the efficiency of anti-inflammatory drugs *in vivo*.

## Introduction

Chemokines, a family of low molecular weight (8–10 kDa) secreted proteins, act as crucial regulators of leukocyte circulation, accumulation, and activation [ 1, 2] . Most chemokines have four typical cysteines, which are classified into C, CC, CXC, and CX3C according to the motif displayed by the first two [3]. Monocyte chemoattractant protein-1 (MCP-1), also named CCL2, belongs to the CC chemokine group because of the location of pivotal cysteine residues that participate in disulfide bonding [ 4, 5] . CC chemokines (CCLs) and their specific CC receptors (CCRs) play crucial roles in pathological and physiological immune cell recruitment [ 3, 6] . MCP-1, a CC chemokine, was initially described as a monocyte chemotactic agent but can also reportedly attract T lymphocytes [ 7, 8] . CCR2, a chemokine receptor for both MCP-1 and CCL7, exhibits a higher binding affinity to MCP-1 [9].

MCP-1 plays essential roles in inflammatory processes, including hepatic inflammation and fibrosis, hepatitis C, autoimmune diseases, cardiac inflammation and fibrosis, chronic neuroinflammation, influenza pneumonia, and sepsis [ 8, 10– 16] . In addition, MCP-1 is significant in many other diseases, such as acute liver injury [17], cirrhotic cardiomyopathy [18], nervous system-related problems [19], osteoporosis [20], insulin resistance [21], diabetic kidney disease [22], polycystic kidney disease [23], macrophage activation syndrome [24], and tumors such as breast cancer [25] and lung adenocarcinoma [26]. Moreover, the infiltration of monocytes into the inflammation site is completely blocked in MCP-1-deficient mice. Although ablation of CCR2 or CCL2 can reportedly attenuate liver damage and fibrosis [ 9, 27] , the extent to which MCP-1 is involved in the *in vivo* pathogenesis, postural distribution, and dynamic inflammatory changes of various diseases remain unclear.

In this study, an MCP-1 luciferase reporter mouse model (MCP-1-Luc) was generated. MCP-1 levels were monitored by capturing luciferase bioluminescence in real-time using an imaging system designed for live mice [ 28, 29] . Our results show that the transgenic MCP-1-Luc mouse is a useful model to study MCP-1 expression in inflammation and disease and to evaluate the efficiency of anti-inflammatory drugs *in vivo*.

## Materials and Methods

### Reagents

Bacterial lipopolysaccharide (LPS), concanavalin A (Con A), corn oil, and dexamethasone were purchased from Sigma-Aldrich (St Louis, USA). CCl _4_ was purchased from Sinopharm Chemical Reagent Co., Ltd (Shanghai, China). Potassium luciferin (Gold Biotechnology, St Louis, USA) was dissolved in phosphate-buffered saline (PBS; Beyotime, Shanghai, China) at 15 mg/mL and stored at –20°C.

### Animals

Mice were placed in a specific pathogen-free environment with a 12/12 h light/dark cycle with food and water provided *ad libitum*. The Institutional Animal Care and Use Committee (IACUC) of Shanghai Engineering Research Center for Model Organisms was responsible for ethical review of all animal study protocols. The IACUC permit number was 2016-0011.

### Generation and genotyping of MCP-1-Luc reporter mice

The firefly luciferase encoding sequence was precisely inserted after an ATG site (translational initiation codon) in the mouse *MCP-1* gene and was therefore driven by the endogenous *MCP-1* promoter. Transgenic mice expressing an MCP-1 reporter on the C57BL/6J background were produced as previously described [30]. Heterozygous transgenic mice were chosen for all experiments unless otherwise stated.

Transgenic founders and their offspring were identified by PCR using the primer set P1/P2. The primer sequences are as follows: P1, 5′-GCCAATTCTTCCCTCTTTCC-3′ (forward) and P2, 5′-GCCT-TATGCAGTTGCTCTCC-3′ (reverse). The positions of P1 and P2 are indicated in Supplementary Figure S1A.

### Acute septic shock model by intraperitoneal (i.p.) injection of LPS

The acute septic shock model was produced by i.p. injection of LPS (3 mg/kg body weight) into mice at the age of 8–12 weeks. Control mice were injected with saline. Luciferase activity was detected through imaging at 0, 1, 3, 5, 8, 12, and 24 h post-injection. At the selected time points after the injection, mice were i.p. injected with luciferin and imaged 12 min later with the Lumazone imaging system as described below. To test the effects of dexamethasone on LPS-induced luciferase expression, mice were co-treated with dexamethasone (3 mg/kg body weight) and LPS, and the control group was injected with saline and LPS. The luciferase signal was monitored through imaging. The mice were sacrificed at 3 h after saline or LPS with or without dexamethasone treatment to detect *MCP-1* mRNA and luciferase activity changes in the heart and liver.

### *Ex vivo* measurement of luciferase activity

Mouse tissues were lysed with 300 μL lysis buffer (Promega, Madison, USA). Luciferase activity was measured using the Luciferase Assay System (Promega) with a luminometer (Lumat LB9507; EG&G, Berthold, Germany). A BCA Protein Assay kit (Beyotime) was used to measure protein concentrations.

### *In vivo* imaging

The Lumazone imaging system (Mag Biosystems, Tucson, USA) was used to perform *in vivo* imaging, as previously described [ 28, 29] . Briefly, the hair on the abdomen of mice was shaved off. Next, mice were administered with 150 μL of potassium luciferin by i.p. injection, anesthetized, and then imaged. Lumazone software was applied to quantify photons emitted from specific areas. Luciferase activity is expressed in photon intensity per second.

### T cell-dependent experimental hepatic injury induced by Con A

Hepatic injury was induced by Con A which was administered by intravenous (i.v.) injection (12 mg/kg body weight) into the tail vein of mice. Mice were given luciferin and imaged at selected time points after treatment. Then mice were anesthetized to collect blood at 5 h and 12 h after Con A administration. Finally, the liver was removed for histological assay and RNA extraction.

### Acute hepatitis and liver fibrosis induced by CCl _4_

In the acute hepatitis group, male mice were administered with CCl _4_ at a dose of 1 mL/kg body weight by i.p. injection. For the fibrosis experiment, the same dosage of CCl _4_ (1 mL/kg) was administered twice a week for 6 weeks. After treatments, mice were photographed at selected time points. At the end of the experiments, mice were euthanized, and samples of the liver were removed for histological examination, hydroxyproline content determination, and extraction of RNA and protein.

### Real-time quantitative PCR

Total RNA was isolated from selected mouse tissues using Trizol (Tiangen, Beijing, China) according to the manufacturer’s instructions and kept at –80°C before use. RNA samples (1600 ng) from mice were reverse transcribed into cDNA using Quant Reverse Transcriptase (Tiangen). SuperReal SYBR Green Premix Plus (Tiangen) was used for qPCR amplification. qPCR was performed on the qTOWER 2.2 (Analytik Jena AG, Jena, Germany). The reaction conditions were as follows: 95°C/ 15 min; 40 cycles of 95°C/ 15 s, X°C/ 20 s, 72°C/ 30 s; 95°C/ 15 s, 60°C/ 15 s. Murine β-actin was used as a reference to normalize the targeted gene expression levels. The sequences of specific primers are listed in Table 1.

**Table:** **Table 1** Sequences of primers used for real-time quantitative PCR

Gene	Forward sequence (5′→3′)	Reverse sequence (5′→3′)
*β-actin*	CCTGTATGCCTCTGGTCGTA	CCATCTCCTGCTCGAAGTCT
*MCP-1*	GGTCCCTGTCATGCTTCTG	GGGATCATCTTGCTGGTGAA
*IL-1β*	GAAATGCCACCTTTTGACAGTG	TGGATGCTCTCATCAGGACAG
*IL-6*	ACAACCACGGCCTTCCCTACT	GCCATTGCACAACTCTTTTCTCAT
*TNF-α*	CCTGTAGCCCACGTCGTAG	GGGAGTAGACAAGGTACAACCC
*iNOS*	GTTCTCAGCCCAACAATACAAGA	GTGGACGGGTCGATGTCAC
*INF-γ*	ATCTGGAGGAACTGGCAAAA	TGAGCTCATTGAATGCTTGG
*Collagen 1*	TAAGGGTCCCCAATGGTGAGA	GGGTCCCTCGACTCCTACAT
*TGF-β1*	CTTCAATACGTCAGACATTCGGG	GTAACGCCAGGAATTGTTGCTA

### Immunofluorescence staining

For immunofluorescence staining, antigen retrieval of deparaffinized tissue sections was performed by microwave on the middle for 20 min. After being blocked with 5% donkey serum (Solarbio, Beijing, China) for 1 h at room temperature, the sections were incubated with primary antibodies at 4°C overnight, followed by incubation with anti-rabbit Alexa Fluor 488 (A21206; Life Technologies, Carlsbad, USA) or anti-goat Alexa Fluor 594 (A11058; Life Technologies) conjugated secondary antibodies. DAPI (Life Technologies) staining was performed for nuclear counterstaining. Fluorescent images were visualized by using a laser-scanning confocal microscope system (OLYMPUS, Tokyo, Japan). The following primary antibodies were used: goat polyclonal anti **-**MCP-1 antibody (sc-1785; Santa Cruz Biotechnology, Dallas, USA) and rabbit polyclonal anti-Firefly Luciferase antibody (ab21176; Abcam, Cambridge, UK).

### Measurement of hepatic hydroxyproline content

A hepatic hydroxyproline assay was performed to quantify collagen content. Briefly, liver tissues were homogenized in lysis buffer at 95°C for 20 min. After the pH value was adjusted to 6.0–8.0, some activated carbon was added to the hydrolysates, followed by centrifugation for 10 min at 2000 *g*. The supernatant was taken to measure the hydroxyproline concentration using a hydroxyproline assay kit (Jiancheng Bioengineering Institute, Nanjing, China) according to the manufacturer’s instructions. Finally, the absorbance was measured at 550 nm and the hydroxyproline level in the hydrolysates was calculated.

### Measurement of the biochemical index in serum

After blood collection, serum was separated by centrifugation at 500 *g* for 15 min at 4°C. Serum aspartate aminotransferase (AST) and alanine aminotransferase (ALT) were tested using an automated chemistry analyser (Sysmex, Tokyo, Japan).

### Western blot analysis

Proteins were extracted from the left lobe using RIPA buffer containing protease inhibitor cocktail (Selleck, Houston, USA), and an equal amount of proteins (70 μg) from each sample was separated by 12% SDS-PAGE and transferred onto PVDF membranes (Millipore, Billerica, USA). The membranes were blocked with Tris-buffered saline and Tween 20 buffer containing 5% fat-free milk powder for 1 h at room temperature and then incubated overnight at 4°C with different primary antibodies, including rabbit monoclonal to alpha smooth muscle Actin (α-SMA; 1:1000; ab32575; Abcam), rabbit polyclonal to TIMP-1 (1:100; sc-5538; Santa Cruz Biotechnology) and Monoclonal Mouse Anti-glyceraldehyde-3-phosphate Dehydrogenase (GAPDH; 1:10000; KC-5G4; Aksomics, Shanghai, China). After extensive wash, membranes were incubated with fluorescein-conjugated secondary antibody for 1 h (1:10000; LI-COR Biosciences, Lincoln, USA). Protein bands of interest were analyzed using the Odyssey Infrared Imaging System (LI-COR). GAPDH was used as the loading control.

### Histological assessment of liver injury

When mice were sacrificed, their liver tissues (left lobes) were collected, incubated in 10% formalin for more than 24 h, and prepared in paraffin blocks according to the standard protocol. The degree of inflammation and tissue damage was observed in paraffin sections stained with hematoxylin and eosin (H&E) using an optical microscope. In the fibrosis experiment, 5 μm-thick liver sections were processed by both H&E staining and Masson’s trichrome staining to assess the architectural alterations and hepatic collagen deposition. Morphometric analysis was then carried out on a computerized image analysis system (Image-Pro Plus; Media Cybernetics, Bethesda, USA). The mean blue-stained area in each section was calculated. The histological severity was graded according to the criteria described by Suzuki *et al*. [31], in which sinusoidal congestion, hepatocyte necrosis, and cytoplasmic vacuolization were graded from 0 to 4.

### Statistical analysis

Data are presented as the mean±SEM. The Bonferroni post hoc test or Brown **–**Forsythe was used for one-way analysis of variance according to the type of data. Comparisons between two groups were assessed by Student’s *t* test. The Mann **-**Whitney U test was used to analyze nonparametric data. *P* values less than 0.05 were considered statistically significant.

## Results

### MCP-1-Luc reporter mouse model was generated by transgenic animal technology

A schematic diagram of mouse model construction is shown in Supplementary Figure S1A. The luciferase coding sequence was precisely inserted at the site of the translational initiation codon ATG in the mouse *MCP-1* gene and was therefore driven by the endogenous *MCP-1* promoter. The transgenic mice were identified by PCR detection of the transgene (using the primer set marked in Supplementary Figure S1A) with tail genomic DNA ( Supplementary Figure S1B) and confirmed by DNA sequencing. This animal model was designated as an MCP-1 expression luciferase reporter mouse, named MCP-1-Luc.

### Induction of luciferase expression in MCP-1-Luc reporter mice by LPS

**Figure 1:**
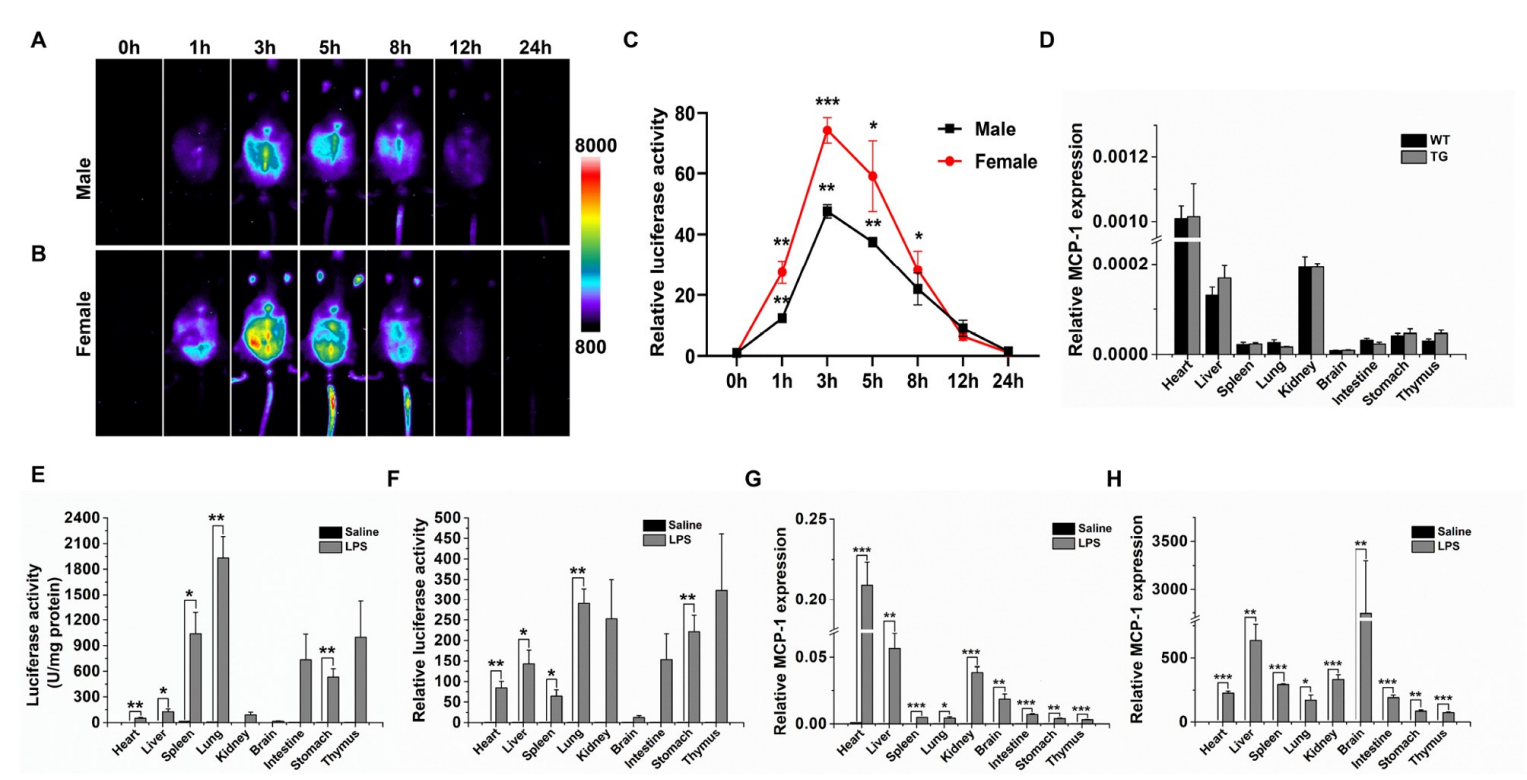
Figure 1 Comparison of the luciferase expression profile and endogenous MCP-1 gene expression in MCP-1-Luc reporter mice following LPS treatment The ventral representative images are diagramed for male (A; n=3) and female (B; n=5) transgenic mice administered with LPS. A color scale indicating intensity/sec is displayed on the right. (C) The changes in LPS-induced luciferase activity are shown as fold changes. (D) MCP-1 gene expression was analyzed by qPCR in male transgenic mice (TG) and wild-type littermates (WT) under normal conditions ( n=4). (E) The luciferase activity in selected organs was measured in the male transgenic mice at 3 h following LPS or saline injection ( n=3). (F) Fold changes of LPS-induced luciferase expression ( n= 3). (G) Endogenous MCP-1 expression in dissected organs was measured by qPCR in male transgenic mice at 3 h after LPS or saline treatment ( n=3). (H) The ratio of LPS-induced endogenous MCP-1 gene expression in selected organs ( n=3). Data are presented as the mean±SEM. * P<0.05, ** P<0.01, and *** P<0.001 vs 0 h (C) or vs saline (E–H).

The mice were validated for luciferase expression in response to LPS injection. Both male and female mice showed significant LPS-induced luciferase signals compared with baseline levels and had a similar tendency of LPS-induced signal changes. The luminescent signal gradually increased and then peaked at 3 h post-injection ( Figure 1A,B). In the male mice, the signal peak was approximately 47-fold of the baseline, and the value was almost 74-fold in females ( *P*=0.0060 and *P*=0.0003, respectively, at 3 h; Figure 1C). The signal gradually decreased to the baseline level at 24 h following LPS treatment ( Figure 1A–C).

### Luciferase expression was induced in parallel with mouse endogenous *MCP-1* mRNA after LPS injection

To confirm the data observed by the *in vivo* imaging system (IVIS), luciferase activity was measured in dissected organs at 3 h after LPS injection. The luciferase activity of the LPS-treated mice was higher in the heart, liver, spleen, lung, kidneys, intestine, stomach, and thymus than that of mice administered saline ( Figure 1E). Compared with saline administration, LPS induced luciferase activity by 84-fold in the heart, 143-fold in the liver, 64-fold in the spleen, 289-fold in the lung, 252-fold in the kidneys, 153-fold in the intestine, 221-fold in the stomach, and 323-fold in the thymus ( Figure 1F). Moreover, endogenous MCP-1 expression was quantified by qPCR ( Figure 1G). The results showed that in parallel with the luciferase activity profile, LPS treatment increased endogenous MCP-1 expression by 226-fold in the heart, 636-fold in the liver, 292-fold in the spleen, 170-fold in the lung, 332-fold in the kidneys, 190-fold in the intestine, 84-fold in the stomach, and 74-fold in the thymus ( Figure 1H). These data indicated that luciferase expression is to a large degree in parallel with endogenous *MCP-1* gene expression in MCP-1-Luc reporter mice. It is worth noting that the MCP-1 expression levels and pattern of distribution in the different organs were similar in wild-type and transgenic littermates ( Figure 1D).

### Luciferase activity in MCP-1 reporter mice was inducible during Con A-induced hepatitis

**Figure 2:**
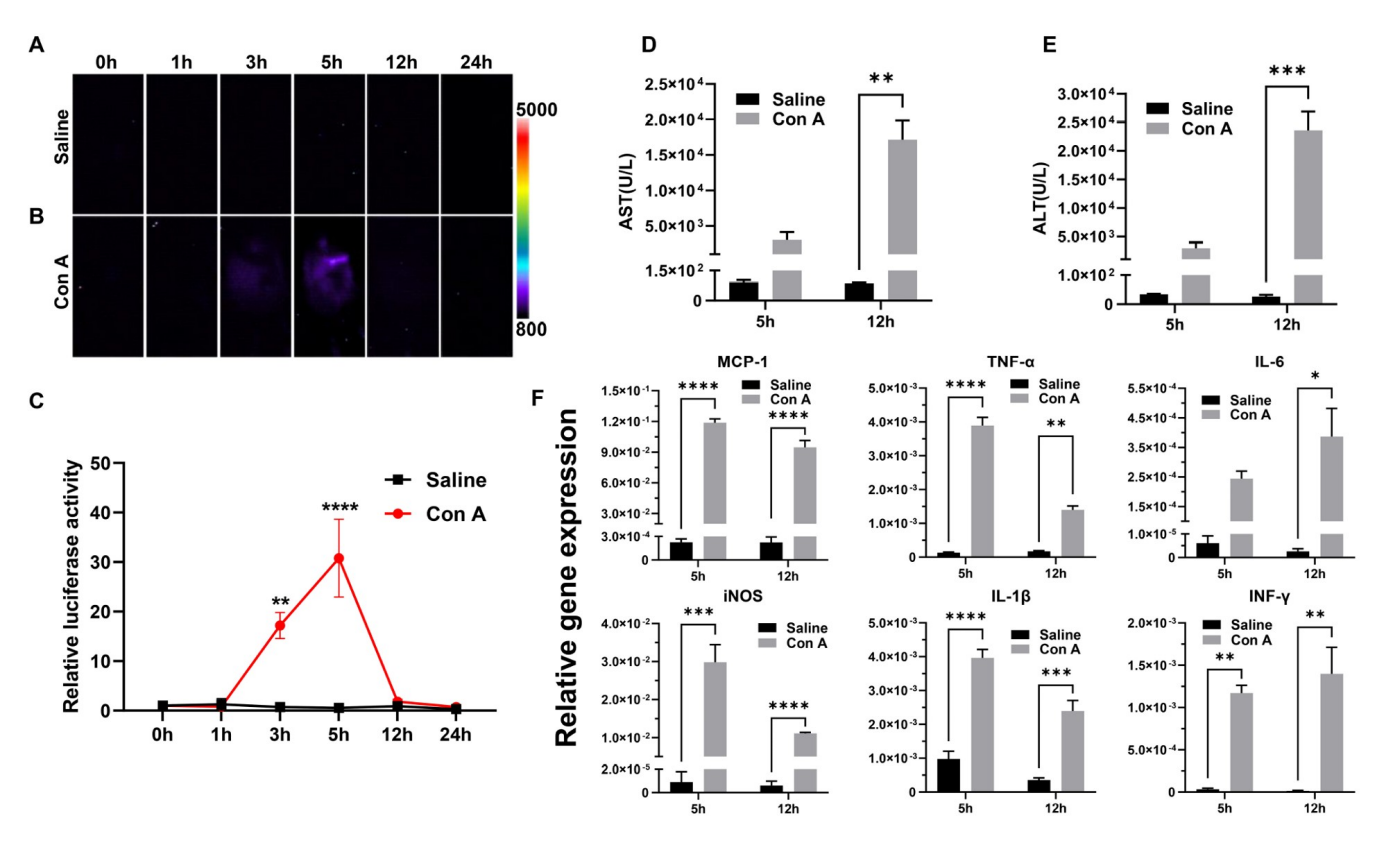
Figure 2 Luciferase expression in MCP-1-Luc reporter mice during Con A-induced T cell-dependent hepatitis Male MCP-1-Luc reporter mice were i.v. injected with saline (A) or Con A (B) and imaged at 0, 1, 3, 5, 12, and 24 h after treatment. Representative images are shown ( n=4). A color scale indicating intensity/sec is displayed on the right. (C) Quantification of luciferase intensity ( n=4). Serum AST (D) and ALT (E) levels were measured ( n=3–6). (F) Relative gene expression levels of MCP-1, TNF-α, IL-6, iNOS, IL-1β, and IFN-γ were measured by qPCR ( n=3–6). Gene expression was normalized to β-actin expression. Data are presented as the mean±SEM. * P<0.05, ** P<0.01, *** P<0.001, and **** P<0.0001 vs saline.

The inducibility of *MCP-1* gene expression in T cell **-**mediated acute hepatic failure was investigated using MCP-1-Luc mice. After i.v. injection of Con A, an abdominal luciferase signal was detected at 3 h, peaked at 5 h, and decreased to baseline in 24 h ( Figure 2A,B). Compared with that in the saline group, the luciferase activity increased 17.2-fold, and 30.8-fold at 3 h and 5 h post **-**Con A injection, respectively ( *P*=0.0018 and *P*<0.0001, respectively; Figure 2C).

Hepatic function was evaluated by measuring serum levels of AST and ALT. AST and ALT levels rapidly increased at 5 h and were further elevated at 12 h following Con A injection compared with the saline group ( *P*=0.0016 and *P*=0.0004, respectively, at 12 h; Figure 2D,E). qPCR was used to quantify the relative gene expressions of *MCP-1*, tumor necrosis factor-alpha ( *TNF-α*), interleukin-6 ( *IL-6*), inducible nitric oxide synthase ( *iNOS*), interleukin-1beta ( *IL-1β*), and interferon-gamma ( *IFN-γ*) in the liver. The results showed that MCP-1, TNF-α, IL-6, iNOS, IL-1β, and IFN-γ expression levels were significantly upregulated following Con A treatment ( *P*<0.05; Figure 2F).

**Figure 3:**
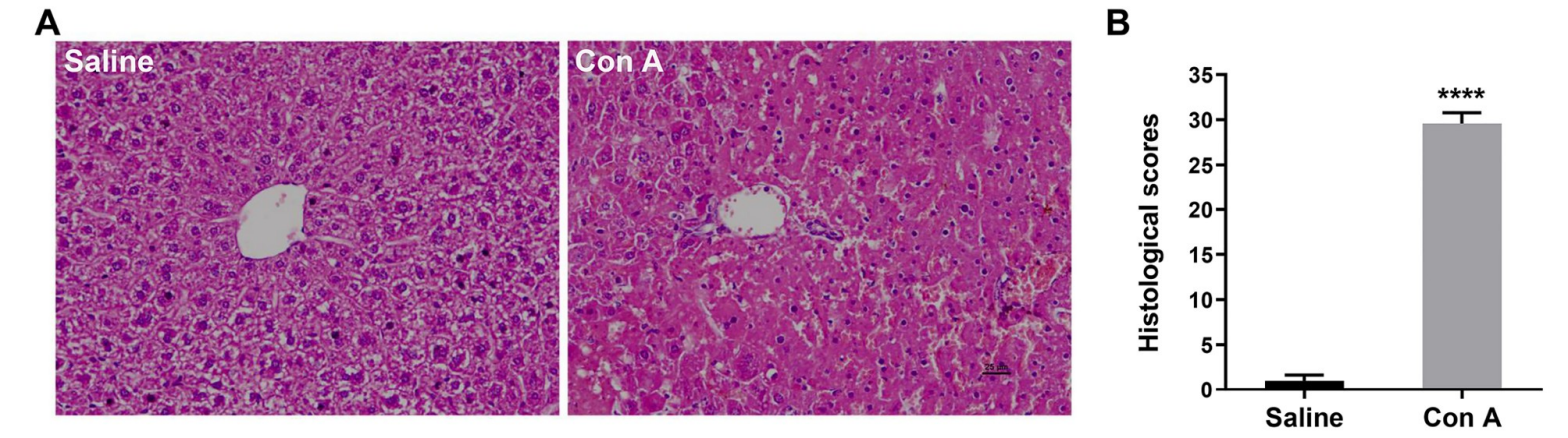
Figure 3 H&E-stained sections of the liver from saline- or Con A-treated mice (A) A representative liver section stained with H&E is shown at 12 h after saline or Con A treatment ( n=3–6). (B) Group averages of Suzuki scores ( n=3–6). Data are presented as the mean±SEM. **** P<0.0001 vs saline. Scale bar =25 μm.

Histopathological changes in liver tissues were observed by H&E staining ( Figure 3A). The structure of liver tissues in the saline control group was completely maintained. In contrast, disordered lobular structures with sinusoidal dilatation and congestion, neutrophil invasion, and ballooning degeneration were observed in mouse livers at 12 h following Con A injection ( Figure 3).

### Luciferase expression in MCP-1-Luc reporter mice during CCl _4_-induced liver damage

**Figure 4:**
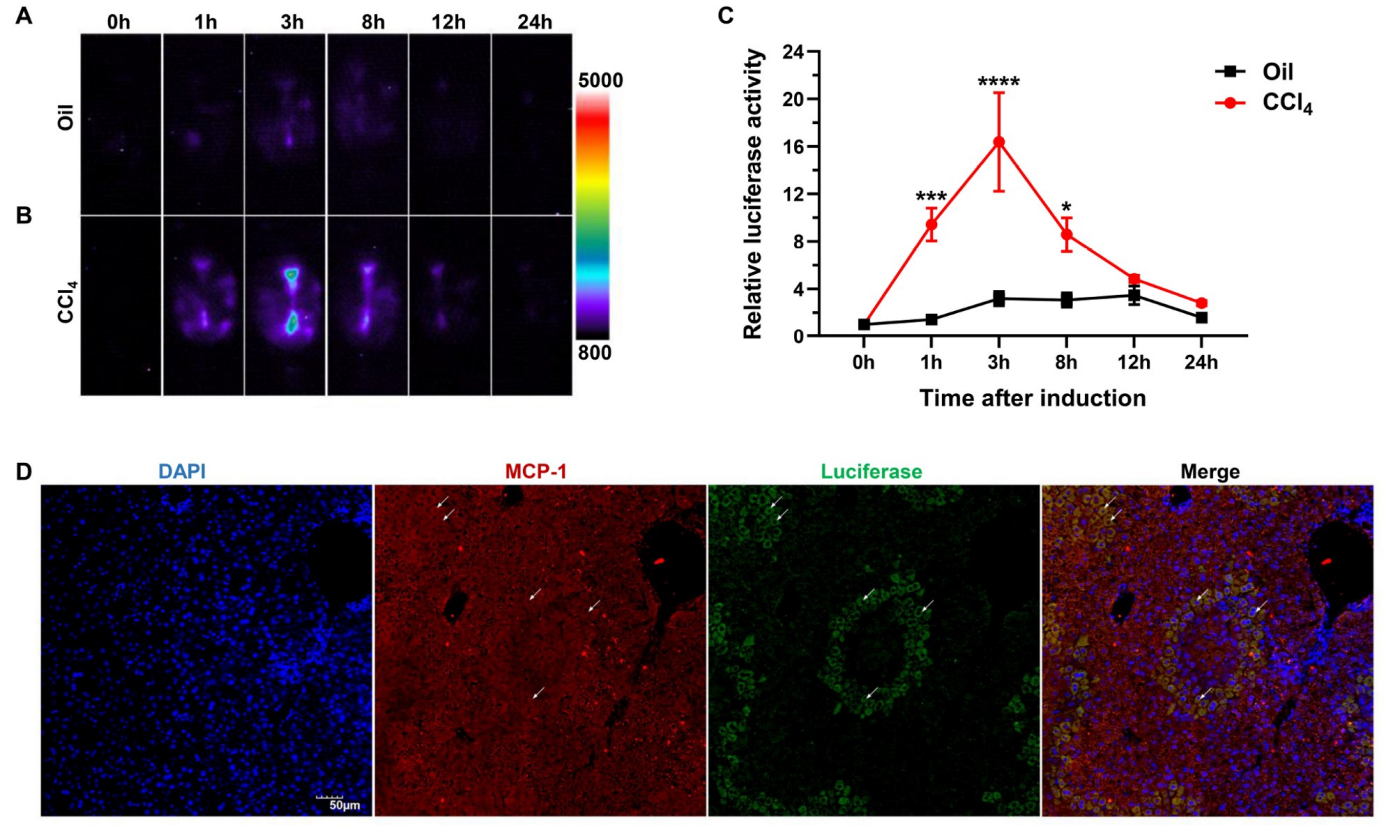
Figure 4 Luciferase expression of MCP-1-Luc reporter mice following CCl _4_-induced acute hepatitis modelling Representative images of male MCP-1-Luc mice were captured at 0, 1, 3, 8, 12, and 24 h after oil (A) or CCl 4 (B) treatment. A color scale indicating intensity/sec is displayed on the right ( n=3–4). (C) Fold changes in CCl 4-induced luciferase intensity ( n=3–4). (D) Co-localization of luciferase with MCP-1 in MCP-1-Luc reporter mice treated with CCl 4 for 3 h was detected by immunofluorescence staining. Blue represents the nuclei; red and green represent MCP-1 and luciferase, respectively. Arrows (white) in the merged image indicate representative co-localization. Data are presented as the mean±SEM. * P<0.05, *** P<0.001, and **** P<0.0001 vs oil. Scale bar =50 μm.

To examine whether MCP-1-Luc reporter mice are suitable for the study of chronic liver injury, fluctuating changes in luciferase intensities in CCl _4_-induced liver damage were examined. Luciferase activity in the abdominal region was detectable at 1 h post **-**CCl _4_ injection during acute hepatitis, peaked at 3 h, and descended to the baseline level at 24 h. Mice administered with solvent oil exhibited a slight increase in luciferase intensity and lower luciferase signals than CCl _4_-treated mice ( Figure 4A,B). Luciferase signals were 9.4-, 16.4-, 8.6-, and 4.8-fold higher than the baseline level at 1, 3, 8, and 12 h, respectively, after CCl _4_ injection ( *P*<0.0001 at 3 h; Figure 4C). To further determine whether the observed changes in luciferase activity mimic endogenous MCP-1 expression, we examined the co-localization of luciferase with MCP-1 in CCl _4_-induced liver injury. Our results showed the co-expression of MCP-1 and luciferase ( Figure 4D).

**Figure 5:**
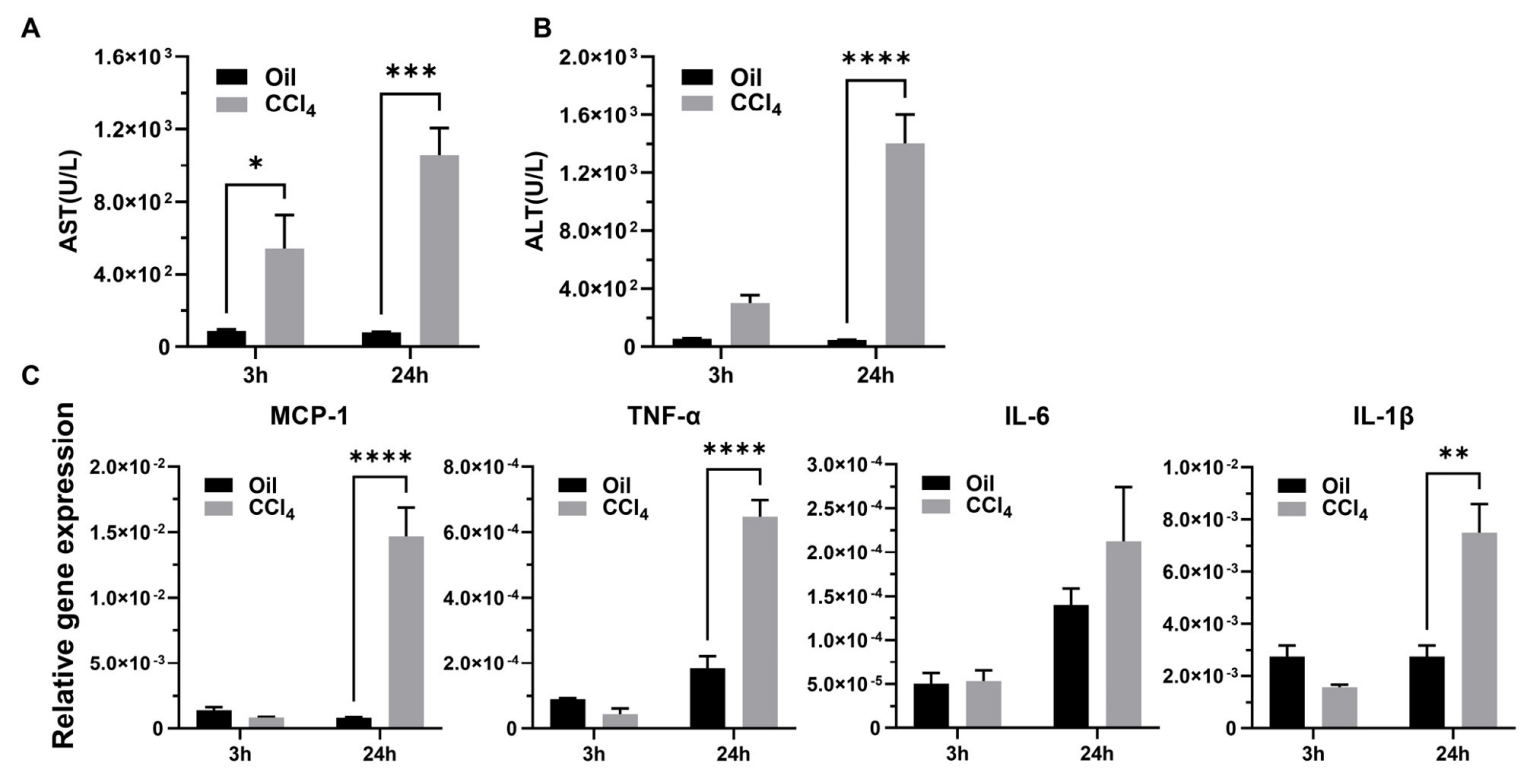
Figure 5 Serum biochemicals and expression levels of MCP-1, TNF-α, IL-6, and IL-1β in MCP-1-Luc reporter mice after CCl _4_ or oil administration for 3 h and 24 h Serum AST (A) and ALT (B) levels were measured ( n=3–4). (C) Quantification of the mRNA levels of MCP-1, TNF-α, IL-6, and IL-1β by qPCR ( n=3–4). Data are presented as the mean±SEM. * P<0.05, ** P<0.01, *** P<0.001, and **** P<0.0001 vs oil.

Hepatic function was evaluated by examining serum AST and ALT levels. Following CCl _4_ injection, serum AST and ALT levels were significantly increased compared with those of the oil-treated group ( *P*=0.0009 and *P*<0.0001, respectively, at 24 h; Figure 5A,B). qPCR-based quantification of *MCP-1*, *TNF-α*, *IL-6*, and *IL-1β* mRNA expression levels in the liver showed that all four were rapidly upregulated by CCl _4_ administration and further increased at 24 h post **-**CCl _4_ injection ( Figure 5C).

**Figure 6:**
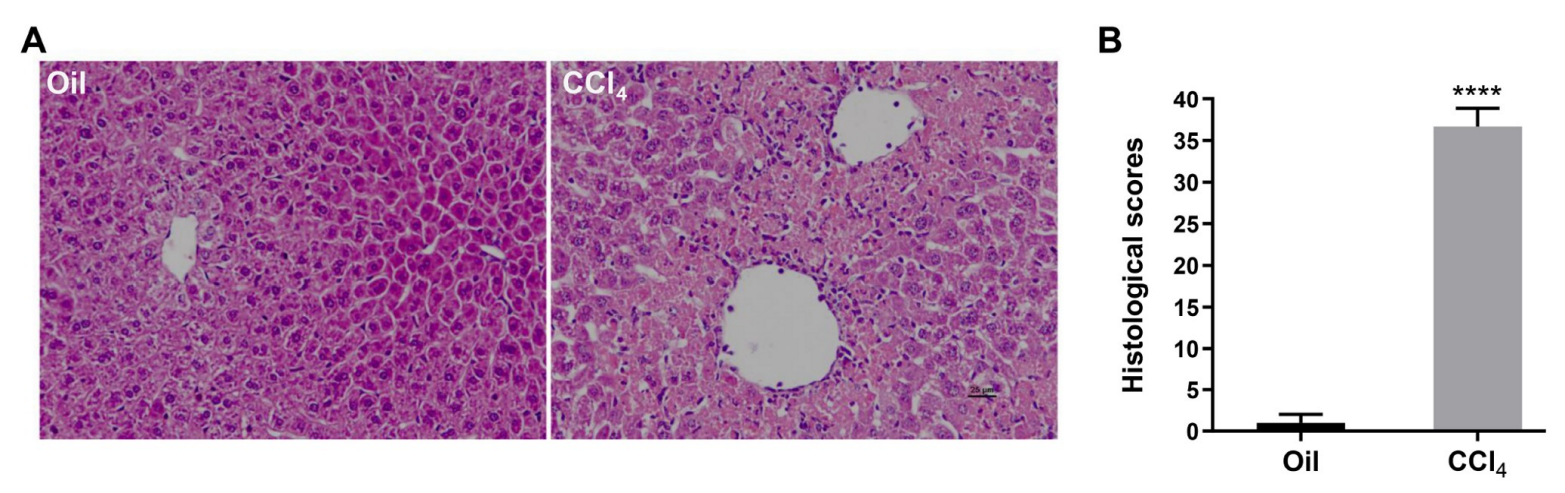
Figure 6 H&E-stained liver sections of mice injected with solvent oil or CCl _4_ (A) A representative liver section stained with H&E is shown at 24 h after oil or CCl 4 treatment ( n=3–4). (B) Histological scores ( n=3–4). Data are presented as the mean±SEM. **** P<0.0001 vs oil. Scale bar= 25 μm.

Histopathological changes in liver tissues were measured by H&E staining. The structure of liver tissues remained normal in the oil-treated group but exhibited injury 24 h after CCl _4_ administration ( Figure 6A,B).

**Figure 7:**
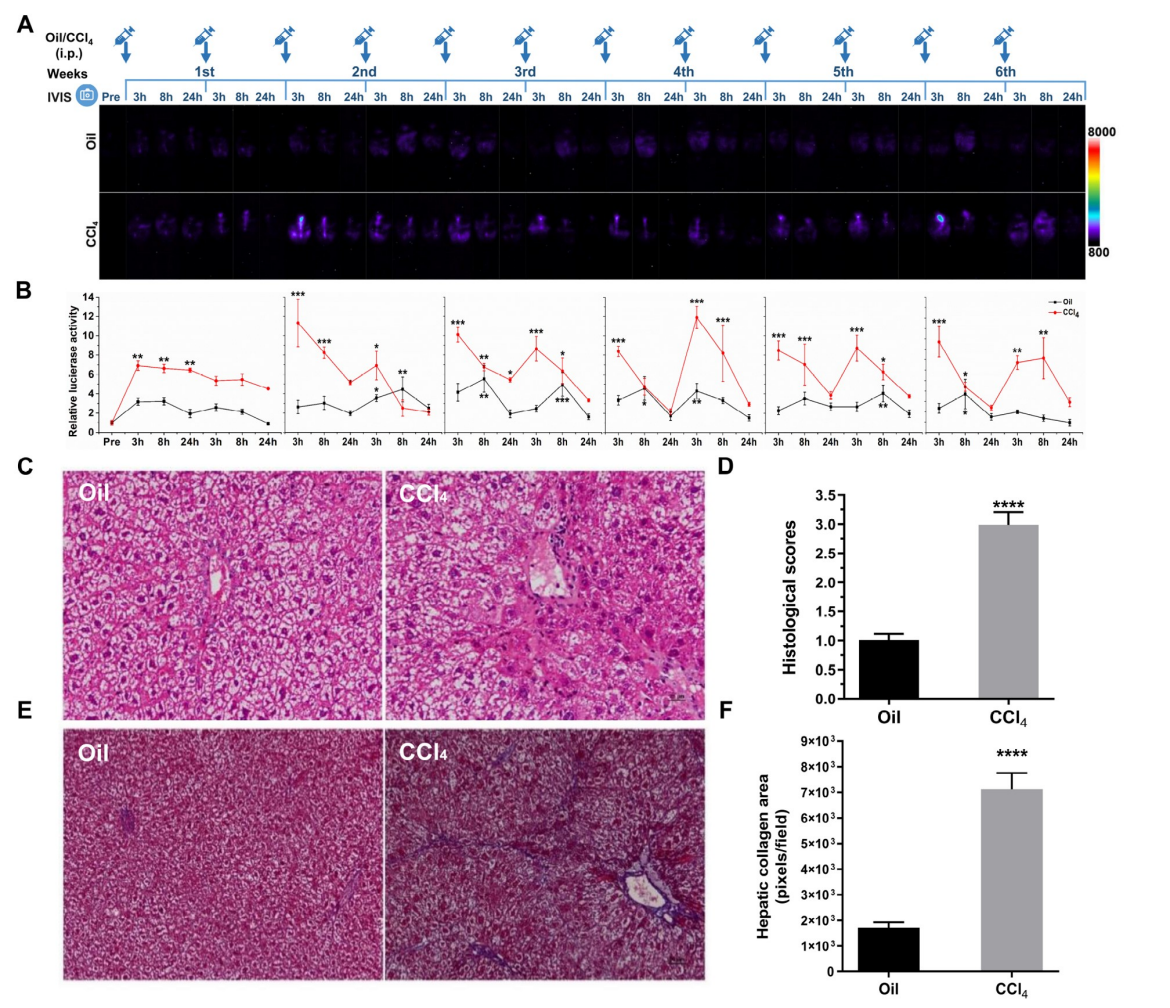
Figure 7 Luciferase expression and histopathological changes in MCP-1-Luc reporter mice during CCl _4_-induced hepatic fibrosis modelling (A) Male transgenic mice were injected with oil or CCl 4 twice a week for 6 weeks, and mice were photographed before (pre) and at 3, 8, and 24 h following each injection. Representative images are shown ( n=3–4). A color scale indicating intensity/sec is displayed on the right. (B) Quantification of luciferase expression during CCl 4-induced hepatic fibrosis modelling ( n=3–4). (C) Representative photos of H&E-stained liver sections and (D) group averages of Suzuki scores ( n=4–5). (E) Collagen deposition by Masson’s trichrome staining and (F) quantitative analysis of the collagen-positive area ( n=4–5). Data are presented as the mean±SEM. * P<0.05, ** P<0.01, and *** P<0.001 vs pre-injection (B), and **** P<0.0001 vs oil (D,F). Scale bars =25 μm for 200×magnification and 50 μm for 100×magnification.

**Figure 8:**
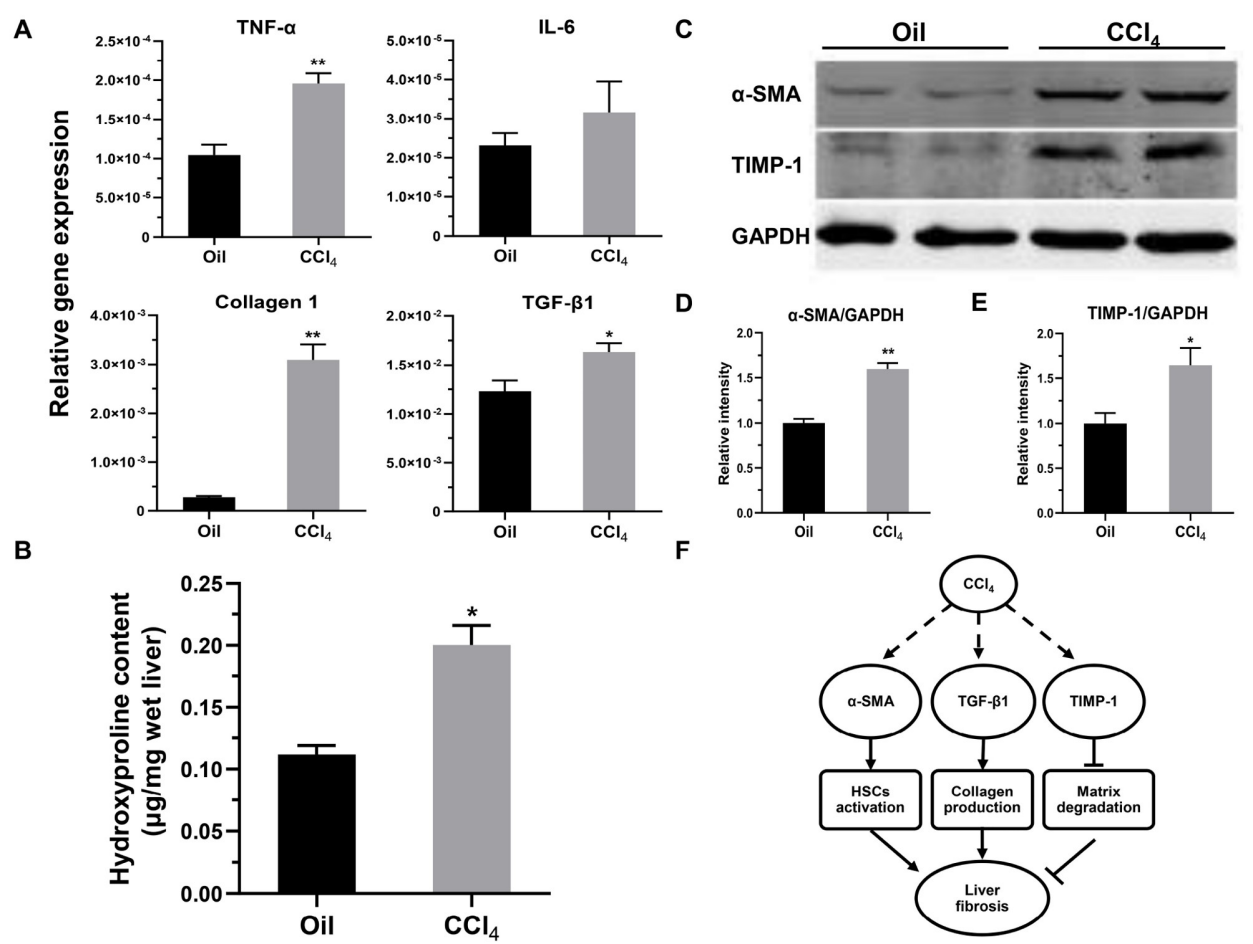
Figure 8 Expression of inflammatory and pro-fibrogenic factors in a CCl _4_-induced hepatic fibrosis model of MCP-1-Luc reporter mice (A) Relative gene expression levels of TNF-α, IL-6, collagen 1, and TGF-β1 were normalized to β-actin expression ( n=4–5). (B) Collagen deposition in liver tissues was quantified by measuring amounts of hydroxyproline (μg/mg liver) after treatments ( n=4–5). (C) Representative western blots and densitometric analyses of α-SMA (D) and TIMP-1 (E) in mice with CCl 4-induced liver fibrosis. Relative intensities were normalized to GAPDH expression, and the mean value for oil-treated mice was set to 1 ( n=4–5). (F) Proposed scheme for the mechanism of CCl 4-induced liver fibrogenesis. Data are presented as the mean±SEM. * P<0.05, and ** P<0.01 vs oil.

Fluctuations in luciferase intensity during CCl _4_-induced liver fibrosis modelling were examined ( Figure 7A). Compared with the oil-administered group, luciferase activity was stimulated and maintained at a high level in the abdomen of mice at 3 h and then declined to a nadir at 24 h after each CCl _4_ treatment ( *P*<0.05 at 3 h; Figure 7A,B). The progression of liver injury was confirmed by Masson’s trichrome and H&E staining at the end of the experiments. We found that the hepatic architecture of CCl _4_-treated mice was damaged, with sinusoidal congestion, neutrophil infiltration, and ballooning degeneration ( Figure 7C,D). Moreover, distinct bridging fibrosis and fibrous septa were observed in liver sections of CCl _4_-treated mice ( Figure 7E,F). qPCR was used to quantify the mRNA levels of *TNF-α*, *IL-6*, *collagen 1*, and *TGF-β1* in the liver. The expressions of these genes were rapidly upregulated in the CCl _4_-administered mice compared with those in the oil-administered mice ( Figure 8A). Furthermore, quantitation of collagen by measuring hepatic hydroxyproline contents also showed that CCl _4_ stimulated fibrogenesis ( *P*=0.0286 vs oil group; Figure 8B). Western blot analysis revealed that molecular factors associated with hepatic stellate cell (HSC) activation (α-smooth muscle actin, α-SMA) and matrix degradation (TIMP-1) were significantly increased ( *P*=0.0024 and *P*=0.0446, respectively; Figure 8C–E). Thus, our results show that CCl _4_ induces liver fibrosis by promoting the expressions of genes involved in fibrogenesis ( Figure 8).

### Dexamethasone inhibited LPS-induced luciferase expression

**Figure 9:**
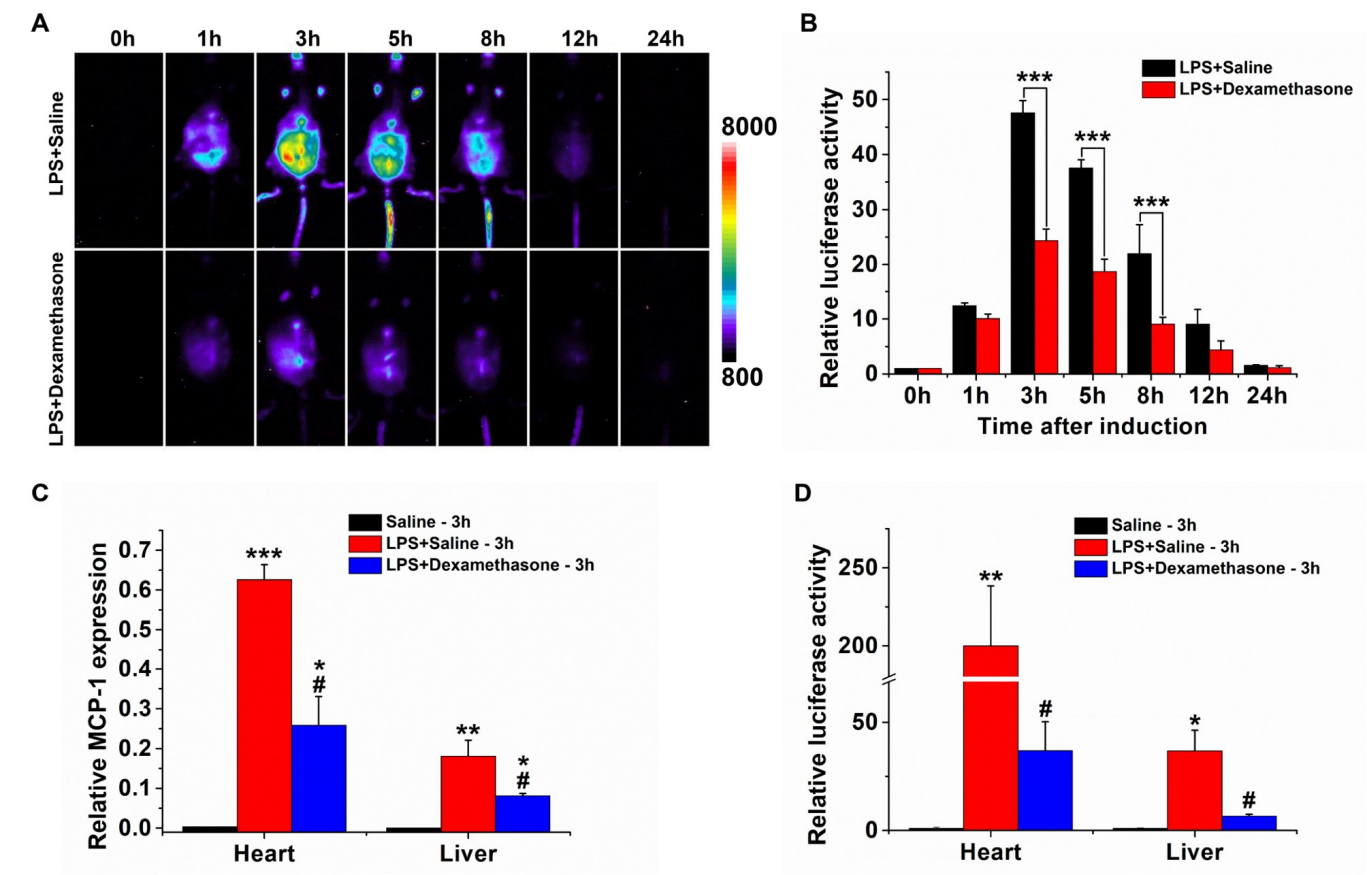
Figure 9 Dexamethasone inhibited the luciferase intensity induced by LPS in MCP-1-Luc reporter mice (A) The luciferase intensity induced by LPS in MCP-1-Luc reporter mice was inhibited by dexamethasone administration. Representative images were captured at 0, 1, 3, 5, 8, 12, and 24 h post-injection ( n=3). A color scale indicating intensity/sec is displayed on the right. (B) Quantification of luciferase activity ( n=3). MCP-1 expression (C) and the relative intensity of luciferase (D) were measured ex vivo in the heart and liver with or without dexamethasone treatment post-LPS injection ( n=3). Data are presented as the mean±SEM. * P<0.05, ** P<0.01, and *** P<0.001 vs LPS+saline (B), or vs saline (C,D); # P<0.05, LPS+dexamethasone vs LPS+saline.

Dexamethasone, a synthetic glucocorticoid, reportedly inhibits LPS-induced immune responses [ 32– 34] . Our results revealed that the luciferase expression induced by LPS was inhibited by dexamethasone administration in MCP-1-Luc reporter mice ( Figure 9A). Compared with baseline luciferase activity, the luciferase signal increased 47-fold at 3 h post **-**LPS injection but only 24-fold in dexamethasone-treated mice ( Figure 9B). LPS-induced endogenous MCP-1 gene expression and luciferase activity in the heart and liver were both significantly inhibited by dexamethasone ( *P*<0.05; Figure 9C,D). These results indicate that MCP-1-Luc reporter mice may be a dependable living model to measure the activity of anti-inflammatory agents *in vivo*.

Thus, we successfully developed a transgenic mouse model that could be a useful model to study transcriptional patterns of MCP-1 expression in inflammation and disease and to evaluate the efficiency of anti-inflammatory agents *in vivo*.

## Discussion

In the current study, an MCP-1 expression reporter mouse, MCP-1-Luc, was established to monitor endogenous MCP-1 expression and analyze the function of MCP-1 during inflammatory processes based on *in vivo* bioluminescence imaging technology. One of the benefits of this technology is that it can be applied to the whole organism with sufficient spatial and temporal resolution to study biological processes *in vivo*. In addition, as a noninvasive, reproducible, standardized and relatively automated method, multiple measurements can be performed on the same living subject at different time points, thus minimizing the number of animals required and experimental cost [35]. Furthermore, it contributes to time and budget reduction in candidate drug selection based on efficacy and safety [ 35– 37] .

We found that the luciferase signals were markedly induced by LPS injection. The results of *ex vivo* trials indicated that luciferase activity responded to LPS treatment in most organs. Moreover, the pattern of luciferase activity matched the changes in endogenous *MCP-1* mRNA expression. Notably, the magnitudes of increased luciferase intensity and endogenous *MCP-1* mRNA expression were not identical in mice, which can be explained by the fact that protein and mRNA expression are not necessarily linearly correlated *in vivo* [38]. In addition, co-localization of luciferase and MCP-1 further suggested that the expression of the luciferase gene is driven by the *MCP-1* promoter.

Con A, a legume lectin, is a mitogen for T cells, monocytes, and other cells [39]. T cell activation is considered to be the preliminary event in viral hepatitis [39]. Hepatitis in mice induced by Con A shares several similar pathologic features with that in humans [40]. Early studies reported that serum MCP-1 level was elevated in hepatitis C patients compared with that in healthy individuals [41]. Endogenous cytokines secreted following Con A administration *in vivo* include TNF-α, IFN-γ, IL-1, IL-2, and IL-6 [ 39, 42, 43] . Among these mediators, IFN-γ and TNF-α play essential roles in the progression of Con A-induced hepatitis [44]. In addition, iNOS-produced NO contributes to the progression of inflammatory hepatic injury [45]. Consistent with these reports, we found that luciferase activity and pro-inflammatory chemokines were induced in the livers of mice following Con A injection, and the expression levels were correlated with the disordered lobular structures as revealed by H&E staining.

The CCl _4_-induced liver damage model, which largely mimics hepatotoxin-induced liver disease in humans, has been used for many years [46]. It has been confirmed that the chemokine-dependent accumulation of monocyte-derived macrophages is a crucial mechanism for persistent hepatic inflammation and contributes to fibrogenesis, both in mouse model trials and human hepatic diseases [ 13, 47] . In this regard, the chemokine receptor CCR2 and its ligand MCP-1 are considered initial factors for the accumulation of inflammatory monocyte subsets in the damaged liver [48]. Hepatocytes, activated macrophages, and HSCs release MCP-1, a chemoattractant that attracts bone marrow-derived monocytes expressing CCR2 [ 49, 50] . Macrophages derived from monocytes maintain a proinflammatory phenotype if liver damage persists. Moreover, HSCs, which play a crucial role in hepatic fibrogenesis, become activated and produce large amounts of collagen during the disease process [51]. In the present study, luciferase signals were monitored during the liver injury process induced by CCl _4_ administration. Luciferase activity associated with acute hepatitis was significantly increased at 1 h after CCl _4_ injection ( *P*=0.0002), before the explosion of pro-inflammatory cytokines and occurrence of tissue lesions, suggesting that MCP-1 upregulation is pivotal to the initiation of hepatitis. Furthermore, our results revealed fluctuations in bioluminescence intensity during hepatic fibrogenesis, indicating that MCP-1 expression is also relevant to liver fibrogenesis induced by CCl _4_. Indeed, the results of the current study are the first to reveal the spatial and temporal distribution of MCP-1 in CCl _4_-induced liver disease.

For decades, a massive number of *in vitro* and *in vivo* experiments have been performed to determine the critical influence of the chemokine system in pathogenic processes of various acute and chronic liver diseases [10]. And it was notable that changes in luciferase signal ( Figures. 2C and 4C) occurred before the appearance of tissue lesions ( Figures. 3A and 6A), implying that the luciferase signal of the MCP-1-Luc reporter mouse model could be used to ascertain the initiation and early progress of liver diseases.

Dexamethasone, a well-known anti-inflammatory drug [32], inhibits both the luciferase signal and endogenous MCP-1 transcription in transgenic mice following LPS treatment. These results demonstrate that the MCP-1-Luc reporter mouse is a sensitive, reliable, and convenient model to monitor MCP-1 gene expression *in vivo* and thus could be used to examine various inflammatory diseases and evaluate the efficacy of anti-inflammatory agents.

Above all, the MCP-1-Luc reporter mouse may be a useful tool not only for tracing MCP-1 expression in a variety of inflammatory conditions but also for investigating the effects of therapeutic drugs on inflammatory diseases.

## Supporting information

22058Supplementary_FigureS1

## Supplementary Data

Supplementary data is available at *Acta Biochimica et Biophysica Sinica* online.
