# Supplementary material for: Bioluminescence imaging of mouse monocyte chemoattractant protein-1 expression in inflammatory processes: Bioluminescence imaging of mouse MCP-1 expression
Source: Acta Biochim Biophys Sin (Shanghai). 2022 Oct 13;54(10):1507–17. doi: 10.3724/abbs.2022143 (PMC9828394; doi:10.3724/abbs.2022143)
Supplement: 22058Supplementary_FigureS1 [file 22058Supplementary_FigureS1.pdf]

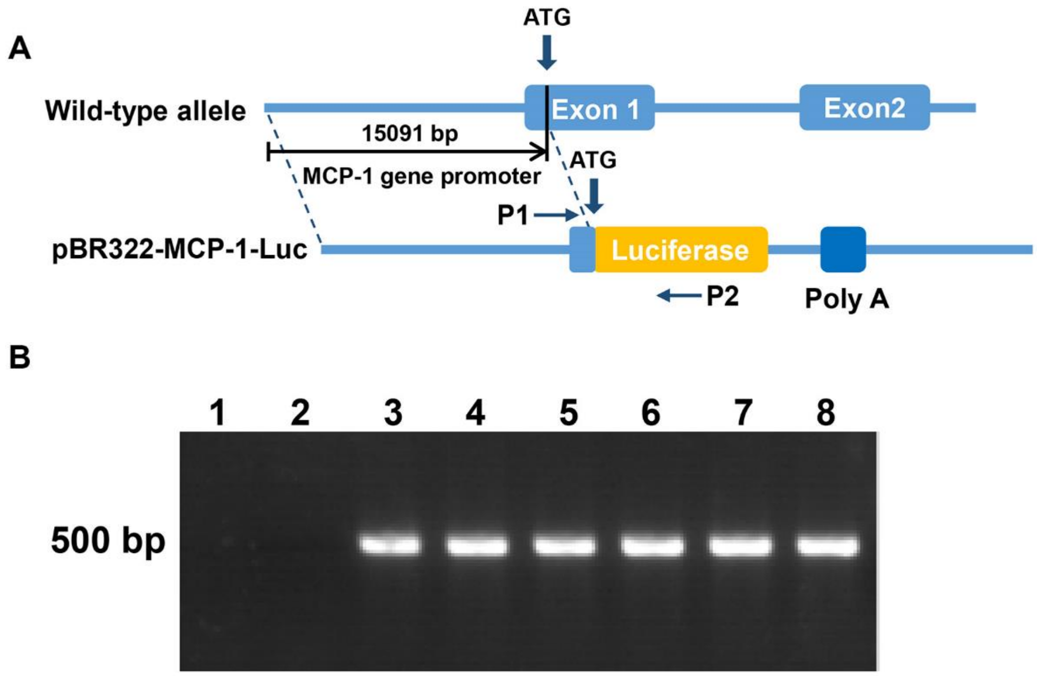

Supplementary Figure S1. Schematic diagram of the transgene structure and PCR strategy of transgenic founder mice screening (A) The MCP-1 luciferase reporter transgene harboring a 15.09 kb mouse *MCP-1* gene promoter and luciferase cDNA was shown. Primers P1 and P2 are indicated. (B) A 500-bp fragment was amplified by PCR in transgenic mice. Lane 1: wild-type littermate; lane 2: ddH_2O; Lanes 3–8: transgenic mice.
